# Book Reviews

**Published:** 1895-02

**Authors:** 


### Book Reviews.

TRANSACTIONS OF THE MEDICAL SOCIETY OF THE STATE OF PENNSYLVANIA, at its Forty-fourth Annual Session, held at Philadelphia, 1894. Volume XXV. Published by the society. Philadelphia: The Edwards & Docker Co., Printers. 1894.

This society clings to the fashion of having annual addresses delivered before it on surgery, medicine, mental disorders, obstetrics, hygiene and ophthalmology—a practice which, at least, seems questionable. We think it would be much better if the time were spent in the reading and discussion of papers on the several subjects.

Among the valuable contributions to this volume may be mentioned one on Chronic Appendicitis, by Dr. John B. Deaver, of Philadelphia; one entitled A Contribution to the Surgery of the Gall-bladder with a Report of Cases, by Dr. X. O. Werder, of Pittsburg, and another on Intra-nasal Surgery, bearing on the subject of Polyps, by Dr. W. H. Daly, of Pittsburg.

There is in an interesting group of papers on Tuberculosis, by Drs. Cooper, Flick, Hamilton, Mays, Munn and others. Among the curious titles we notice Christian Science and its Relation to the Medical Profession, by Dr. H. H. Longsdorf, of Carlisle, and Should the Journal of the American Medical Association be Used to Promote Quackery? by Dr. S. Solis Cohen, of Philadelphia.

There is an absence of reported discussions in this volume which we think detracts from its value, but the papers published in it are, for the most part, of excellent merit.

---

TEXT-BOOK OF ANATOMY AND PHYSIOLOGY FOR NURSES. Compiled by DIANA CLIFFORD KIMBER, Graduate of Bellevue Training School; Assistant Superintendent New York City Training School, Blackwell's Island, N. Y. Octavo, pp. xvi.—245. Price, $2.50. New York and London: Macmillan & Co. 1894.

Anatomy and physiology furnish the foundation for the study and proper understanding of medicine; likewise, it is the basis for the proper instruction of all nurses who submit themselves to that adequate training necessary to fit themselves for the practice of their profession. The proper understanding of anatomy and physiology can only be acquired by the medical student in the dissecting room and laboratory; but the trained nurse does not require the minute details of these subjects that are essential for the practice of medicine.

This is the first attempt, in so far as our knowledge extends, to compile a text-book on anatomy and physiology for the special use of nurses. Heretofore it has been necessary to extract from general treatises that which is applicable—a labor requiring much expenditure of time and no end of trouble.

This author demonstrates her experience in an unmistakable manner through the eminently practical work that she has compiled. Its arrangement is admirable, and she has blended the two subjects with excellent cohesion. She has also illustrated the book with good judgment. The chapters on the tissues are to be specially commended. It is a book that should find its way into the hands of every nurse.

---

A DICTIONARY OF MEDICINE, including General Pathology, General Therapeutics, Hygiene and the Diseases of Women and Children, by various writers. Edited by RICHARD QUAIN, Bart., M. D., London, LL. D., Ed., F. R. S., President of the General Council of Medical Education; Member of the Senate of the University of London; Hon. M. D. Trinity College, Dublin; Hon. M. D. Royal University of Ireland; Fellow, late Vice-President and Senior Censor of the Royal College of Physicians; Hon. Fellow of the Royal College of Physicians of Ireland; Physician Extraordinary to Her Majesty the Queen; Consulting Physician to the Hospital for Diseases of the Chest, Brompton, and to the Seamen's Hospital, Greenwich. Assisted by Frederick Thomas Robarts, M. D., London, B. Sc., and J. Mitchell Bruce, M. A., Aberdeen, M. D., London, with an American Appendix, by Samuel Treat Armstrong, M. D., Ph. D., Visiting Physician to the Harlem, Willard Parker and Riverside Hospitals, New York, etc. New edition, revised throughout and enlarged. Imperial 8vo, in two volumes, pp. 2564, illustrated. New York: D. Appleton & Company. 1894.

Twelve years ago, when the first edition of Quain's Dictionary appeared, it at once met the favor of the profession, and the review columns of the medical journals were full of praise for the manner in which the editor had grouped such a vast amount of information and made it available for ready reference. It is asserted, since its first appearance, that more than 33,000 copies have been issued. This, alone, furnishes substantial proof of the value of the work.

The motive of the editor was a desire to place in the hands of the practitioner of medicine, as well as the teacher and student, a means of ready reference to the accumulative knowledge which we possess of scientific and practical medicine. Progress, of late, has been so rapid, literature so voluminous, and much of it so fragmentary and scattered, that the labor of putting it into accessible shape has been enormous. That, however, it has been done most satisfactorily must be apparent to everyone who will critically examine this latest edition.

The work, now, consists of two volumes, and has been entirely reprinted in larger type. The number of pages has been increased from 1834 to 2566, and forty-three new illustrations have been added, increasing their total number to 181.

It is, by far, the most complete dictionary of medicine in two volumes extant. The mechanical execution of the work ranks among the best, and the continued favor of the profession—practitioners, teachers and students,—is already assured.

THE PROCEEDINGS of the Fourth Annual Meeting of the Association of Military Surgeons of the United States. Held at Washington, D. C., on May 1, 2 and 3, 1894. Octavo, pp. 816. St. Louis: Buxton & Skinner Stationery Co. 1894.

The meeting of this association at Washington last year, as recorded in this volume, must have been an excellent one, for the book is full of interesting papers. The president, Dr. N. Senn, of Chicago, as the subject of his annual address, discoursed upon Abdominal Surgery on the battlefield. If our present knowledge of aseptic methods in surgery had been acquired prior to the civil war, many lives could have been saved by promptly opening the abdomen, suturing the visceral wounds, irrigating the cavity and a discreet employment of drainage. It is the province of this association to thoroughly instruct its members on this important branch of military surgery, and Dr. Senn has performed good service in taking the initiative in this matter.

The second paper relates to the transportation of wounded in war, and is written by Dr. Charles Smart, of the army, who was one of the most efficient field medical officers in the second army corps during the war of the rebellion. His paper, as might be anticipated, is both interesting and instructive. But we might go on through the book and make similar comment on many of the papers that it contains. Unfortunately, however, we have neither time nor space in which to do justice to the book.

The association is a well-organized and disciplined body that is doing most excellent work. It will meet in Buffalo May 21, 22 and 23, 1895, under the presidency of Dr. George M. Sternberg, surgeon-general of the United States army. Dr. Albert H. Briggs, surgeon of the 65th Regiment, N. G., S. N. Y., is chairman of the committee of arrangments, which is a guaranty that the association will be amply provided for during its visit at Buffalo.

---

DISEASES OF THE CHEST, THROAT AND NASAL CAVITIES. Including Physical Diagnosis and Diseases of the Lungs, Heart and Aorta, Laryngology and Diseases of the Pharynx, Larynx, Nose, Thyroid Gland and Esophagus. Third edition, revised. With 240 illustrations, 8vo, 718 pages. By E. FLETCHER INGALS, A. M., M. D., Professor of Laryngology and Practice of Medicine, Rush Medical College; Professor of Diseases of the Throat and Chest, Northwestern University Woman's Medical School, etc., etc. Price, muslin, $5.00. New York: William Wood & Company. 1894.

The appearance of a third edition of this work so soon after the second edition was issued, strongly accentuates the favor with which the treatise is received by the profession. There is every reason why diseases of the chest, throat and nasal cavities should be considered in one treatise, since very many diseases of the chest take origin in the upper air tract. Again, many supposed diseases of the lungs are really not so, but only simulated, the symptoms being reflexes from affections higher up, in the throat or nose.

Ingals is an experienced teacher, a methodical student and a reliable author. He does not waste time with the discussion of questionable theories or doubtful methods of treatment. His work, therefore, commends itself to the general practitioner of medicine as a safe guide to the understanding of the diseases which it considers, and to the specialist as a practical working exposition. A few pages have been added to this edition, in order to keep it abreast of scientific medical progress, and an appendix contains many valuable formulæ.

A copious index serves to complete one of the best treatises of its kind.

---

A MANUAL OF MODERN SURGERY, General and Operative. By JOHN CHALMERS DA COSTA, M. D., Demonstrator of Surgery, Jefferson Medical College, Philadelphia; Chief Assistant Surgeon Jefferson Medical College, etc. Saunders's New Aid Series. Small 8vo, pp. 809, with 188 illustrations in the text and thirteen full-page plates in colors and tints. Price, $2.50 net. Philadelphia: W. B. Saunders, 925 Walnut street. 1894.

The author of this work announces that it seeks to stand between the text-book and compend, and we are of the opinion that this is a proper estimate of its true place in surgical literature. The text-books must needs be studied and consulted by every surgeon worthy of the name, no matter how cumberous it may seem; while the student who only aims at superficiality will be contented with a compend, no matter how incomplete it may be. Both these opposite and divergent classes may meet on common ground in the study of Da Costa's excellent manual.

Here the student will find that obsolete and unessential methods have been excluded, to the better understanding of those that are in vogue in the present day. Bacteriology is considered in the opening chapter, and then inflammation, that subject which is so little understood and so greatly misunderstood, is intelligently discoursed upon in the next forty pages. These two subjects, together with tubercular diseases of bone, deserve profound study by every person who makes any attempt to practise surgery.

Very wisely, we think, surgical questions relating to ophthalmology, gynecology, rhinology, otology and laryngology have been eliminated from this work, the author very modestly and properly conceding that only the specialist is competent to write upon each of these branches. For like reasons, only the practical questions in orthopedic surgery are discussed, such as hip disease, club-foot and Pott's disease of the spine. On the other hand, very considerable space is allotted to consideration of fractures and dislocations, these subjects being of paramount practical importance.

How deficient in knowledge, with reference to the diagnosis and treatment of fractures and dislocations, the average practitioner of medicine is, can be verified by an examination of his results. The fact is, only a small proportion of physicians, who are regular graduates in medicine, ever become thoroughly competent to treat these injuries.

Operative surgery receives important and elaborate consideration, and a few brief pages on asepsis and antisepsis close the volume. The index is voluminous, the illustrations are generally good and the mechanical execution of the volume is excellent.

In this brief notice we have only pointed out a few special points that this work possesses. It is an excellent manual, carefully written by a talented teacher and skilful surgeon, who in it has most satisfactorily made his début as an author.

---

TRAVAUX D'ELECTROTHERAPIE GYNECOLOGIQUE. Archives Lemestrielles D'Electrothérapie Gynécologique Fondées et Publiés. Par le Dr. G. APOSTOLI. Vice-Président de la Société Francaise D'Electrothérapie, etc. Volume Fascicules I. et II. Paris. 1894.

This work was founded and published by Apostoli for the purpose of grouping all papers and clinical reports appearing in the several countries, to provide gynecologists with a book of reference concerning the employment of electricity in this branch of medicine.

This first volume contains papers taken from English, Belgic, American, Russian, Austrian, German, Polish, Danish, Hungarian and Italian literature.

Apostoli pays tribute to Dr. A. Tripier, whom he credits with being the father of the essentially French science of gynecological electro-therapeutics. This first volume, containing 711 pages, is minute in detail and will be useful as a book of reference. At the end is a bibliography of the subject.

---

A PRACTICAL TREATISE ON MATERIA MEDICA AND THERAPEUTICS, with Especial Reference to the Clinical Application of Drugs. By JOHN V. SHOEMAKER, A. M., M. D., Professor of Materia Medica, Pharmacology, Therapeutics, and Clinical Medicine, and Clinical Professor of Diseases of the Skin in the Medico-Chirurgical College of Philadelphia; Physician to the Medico-Chirurgical Hospital; Member of the American Medical Association, of the Pennsylvania and Minnesota State Medical Societies, the American Academy of Medicine, the British Medical Association; Fellow of the Medical Society of London, etc., etc. Second edition, revised. Volume II., 680 pages. An independent volume upon Drugs. Price, in cloth, $3.50 net; sheep, $4.50 net. Philadelphia: The F. A. Davis Company, publishers, 1914 and 1916 Cherry street. 1893.

This volume was received some time ago, and its notice delayed through inadvertence. It is entirely devoted to the consideration of drugs, as its title indicates. Brief accounts are given of some of the recent additions to materia medica, some of which consist of the newer drugs, and others are animal extracts and juices, as well as the inoculation protectives, tuberculin and tuberculocidin.

The recent progress making in the use of these latter agents indicates that infection, in many virulent and fatal diseases, must be neutralized by bacterial products. This conviction is increasing more and more constantly as time advances, and seems to be sustained by experiences now making with the diphtheria antitoxin. It is probable, therefore, that whether this latter agent should prove successful or not in controlling diphtheria, some other like bacterial product will be discovered of potent agency in neutralizing toxemia resulting from the infection.

The description of drugs in this book is concise and their pharmacology and therapy satisfactorily condensed without curtailing their comprehensiveness. It is a work in which the practitioner of medicine will find much satisfaction, because in it the application of drugs to the treatment of disease is kept constantly uppermost. Students, too, will find it a most excellent working treatise.

---

A MANUAL OF THE PRACTICE OF MEDICINE, prepared especially for Students. By A. A. STEVENS, A. M., M. D., Lecturer on Terminology and Instructor in Physical Diagnosis in the University of Pennsylvania; Demonstrator of Pathology in the Woman's College, Philadelphia; Physician to St. Mary's Hospital, etc., etc. Third edition, revised. Illustrated. Small 8vo, pp. xviii.—501. Price, $2.50. Philadelphia: W. B. Saunders, 925 Walnut street. 1894.

The third edition of this work appears within two years after its first publication. This would seem to indicate its popularity. There is no doubt that in the present day a tendency to superficial knowledge is one of the evils that we constantly meet, and it would appear that such a book as this tends to foster superficiality in teaching or in the study of medicine. Stevens, however, has prepared one of the best manuals of its kind, and it could be advantageously employed as an index to the general subject of internal medicine. It serves to refresh the well-trained and fully-equipped mind, but does not take the place of the complete treatise.

We congratulate the author on the success he has attained.

---

LANDMARKS IN GYNECOLOGY. By BYRON ROBINSON, B. S., M. D., Chicago, Ill.; Professor of Gynecology in the Chicago Post-Graduate School; Gynecologist to the Woman's Hospital, the Charity Hospital and the Post-Graduate Hospital. The Physicians' Leisure Library Series. In two volumes. Price, cloth, 50 cents; paper, 25 cents. Detroit, Mich.: George S. Davis. 1894.

These readable little books contain an abstract of some of the author's lectures delivered at the Chicago Post-Graduate Medical School in recent years. The landmarks in gynecology which he considers of chief importance are anatomy, menstruation, labor, abortion, gonorrhea and tumors.

Robinson, who is an easy writer, is especially strong in his consideration of gonorrhea. The dangers, he says, from gonorrhea in women arise from the spread of the infection to the peritoneum and kidney. He describes it as an infectious disease produced by the gonococcus. This germ may pass, he continues, from the vulva through the vagina to the cervix, from whence it may extend over the endometrium and through the Fallopian tube to the peritoneum, where it gives rise to plastic peritonitis, suppurative peritonitis and ovaritis, which may end in recovery, but often in invalidism or death.

Robinson uses the diphthong in certain words, like gynecology and gonorrhea, while he omits it in spelling certain other words. We always omit them wherever practicable. We see no reason why they should be used in gynecology and gonorrhea and omitted from peritoneum and hemorrhage.

These little books deserve to be carefully read by general practitioners of medicine.

---

BOOK OF THE NEW YORK STATE REFORMATORY, Elmira, N. Y. Eighteenth year. Containing the Annual Report of the Board of Managers for the year ending September 30, 1893. Transmitted to the State Legislature, January, 1894.

This report is handsomely printed on highly-finished heavy book-paper, and is illustrated by a number of well-executed photogravures and half-tones. It is a history of the scholastic, military and financial status of the Reformatory for a year, and in its reading is found one of the best of arguments for a continuation of confidence in the management.

The recent ordeal through which it has passed accentuates the opinion which we here express,—namely, that there are no persons as fit to judge of the needs of the institution, and of the proper conduct of its superintendent, as the managers themselves.

This handsome book is printed at the Reformatory.

---

ESSENTIALS OF DISEASES OF THE SKIN, including the Syphilodermata, Arranged in the form of Questions and Answers prepared especially for Students of Medicine. By HENRY W. STELWAGON, M. D., Ph. D., Clinical Professor of Dermatology in the Jefferson Medical College; Physician to the Department of Skin Diseases, Howard Hospital, etc., etc. Saunders's Question Compends No. 11. Third edition, revised and enlarged, with seventy-one letter-press cuts and fifteen half-tone illustrations. Philadelphia: W. B. Saunders, 925 Walnut street. 1894.

This is a most excellent condensation of the essentials of diseases of the skin, and will serve to remind the student of the leading characteristics of the most common maladies in dermatology. The illustrations have been improved over the former edition, and other additions made keep it abreast of the science.

THE JOHNS HOPKINS HOSPITAL REPORTS. Report in Surgery II., Volume IV. No. 6. The results of operations for the cure of cancer of the breast performed at the Johns Hopkins Hospital from June, 1889, to January, 1894. By WILLIAM S. HALSTED, M. D., Professor of Surgery Johns Hopkins University ; Surgeon-in-chief to the Johns Hopkins Hospital. Baltimore: The Johns Hopkins Press. 1894.

In this brochure the author records fifty cases operated on for the cure of cancer of the breast, by what is called the complete method, in which he has been able to trace only three local recurrences. Great interest, of late, attaches to this operation, in which surgeons all over the world are endeavoring to discover some method of preventing a return of the disease. Halsted describes his cases with much detail, and surgeons will examine the work with considerable interest.

---

INTERNATIONAL CLINICS. A Quarterly of Clinical Lectures on Medicine, Neurology, Pediatrics, Surgery, Genito-urinary Surgery, Gynecology, Pharyngology, Rhinology, Ophthalmology, Laryngology, Obstetrics, Otology and Dermatology. By professors and lecturers in the leading medical colleges of the United States, Great Britain and Canada. Edited by JUDSON DALAND, M. D., (Univ. of Penna.,) Philadelphia, Instructor in Clinical Medicine and Lecturer on Physical Diagnosis in the University of Pennsylvania; Assistant Physician to the University Hospital ; Physician to the Philadelphia Hospital and to the Rush Hospital for Consumptives. J. Mitchell Bruce, M. D., F. R. C. P., London, England, Physician and Lecturer on Therapeutics at the Charing Cross Hospital. David W. Finlay, M. D., F. R. C. P., Aberdeen, Scotland, Professor of Practice of Medicine in the University of Aberdeen ; Physician to, and Lecturer on, Clinical Medicine in the Aberdeen Royal Infirmary ; Consulting Physician to the Royal Hospital for Diseases of the Chest, London. Volume III Fourth series. 1894. Royal 8vo, pp. xii.—363. Philadelphia : J. B. Lippincott Co. 1894.

This book is one of the best of the series. It is marked by an increase in illustrations, containing more plates and figures than any of its predecessors, showing a disposition on the part of the publishers and contributors to take heed of the criticisms heretofore expressed in this regard.

The Buffalo contributors to this volume are Dr. Charles G. Stockton, on Chorea ; Dr. Roswell Park, on Perineal Section following Suprapubic Cystotomy, and Hernia ; and Dr. M. D. Mann, on An Intraligamentary Cyst.

Dr. Thomas Hunt Stucky, of Louisville, contributes an interesting lecture on Disorders of Digestion ; Dr. C. B. Burr, of Flint, Mich., speaks instructively on Forms of Insanity ; Dr. V. P. Gibney, of New York, discourses on Congenital Dislocation of the Hip ; Dr. John B. Hamilton, of Chicago, delivers a multiple lecture entitled Traumatic Necrosis,—Various forms of Tuberculosis —Epithelioma of the Lip,—Multilocular Hydrocele ; and Dr. L.

H. Dunning, of Indianapolis, adds to the interest of the volume in a fine lecture on Fibroma of the Fallopian Tubes,—Pyosalpinx,—Laparatomy,—Acquired Atresia of the Vagina.

There are other subjects of professional interest dealt with intelligently in this book, but the specimens we give convey an idea of the versatility of the volume.

---

TRANSACTIONS OF THE MICHIGAN STATE MEDICAL SOCIETY for the year 1894. Volume XVIII. Detroit: Published by the Society. 1894.

Among the many volumes of state medical society transactions issued during the year 1894, that of Michigan must necessarily take a foremost place. This judgment must stand whether grounded upon the excellence of the papers read, the quality of the discussions, or the way and manner in which the volume is edited and printed.

The accomplished president, Dr. Eugene, Boise of Grand Rapids, took for the subject of his annual address, Organization, a Necessity for Professional Progress, in which he advocates the uniting the state societies with the national association in such a way as to make the presidents of each state society executive lieutenants in the national body. So, too, would the officers of each county society become subordinates of the state societies. This plan already exists in the state of New York, where the county societies hold allegiance to the State society, in which they are annually represented by delegates.

We notice many familiar names among the officers, members of committees, and Fellows of this society. Dr. Henry O. Waker, of Detroit, is the president for 1895, and the next annual meeting will be held at Bay City during the first week in June.

---

TEXT-BOOK OF HYGIENE: A Comprehensive Treatise on the Principles and Practice of Preventive Medicine from an American Standpoint. By GEORGE H. ROHE, M. D., Professor of Therapeutics, Hygiene and Mental Diseases, in the College of Physicians and Surgeons, Baltimore; Superintendent of the Maryland Hospital for the Insane; Member of the American Public Health Association; Foreign Associate of the Sociétè Française d'Hygiéne, etc. Third edition, thoroughly revised and largely rewritten, with many illustrations and valuable tables. Royal 8vo, 553 pages. Cloth, $3.00 net. Philadelphia: The F. A. Davis Co., 1914 and 1916 Cherry street. 1894.

The increasing interest in preventive medicine is testified to by the additions to the text-book literature that are constantly being made in all countries. Nowhere is this more evident than in the United States, where, of late, these books have not only been increased in quantity but decidedly in quality.

The author of this treatise is well known to the professional world as a teacher of distinction and a scientist of learning. Since the first edition of his book appeared, sanitary science has increased in a remarkable degree, hence Professor Rohé, not to be outstripped in the race, has revised his text-book in every chapter. That he has succeeded in bringing it well abreast of the advanced condition of sanitary science is apparent upon even a casual examination, while a more minute scrutiny develops the fact that it has become one of the best text-books that has been published in this country.

Attention, of late, has been called to the subject of quarantine, and recent legislation has very much changed the practice on this side of the Atlantic. In this work, special attention is given to this subject as well as to its kinsman, marine hygiene.

In order to make the book of still greater value to teachers, students and sanitary officers, the author has appended to each chapter an analytical set of questions, while a separate section has been added on the examination of air, food and water.

Taken altogether, it is one of the most satisfactory text-books yet offered to the medical colleges, and we hope to see it universally accepted as a teaching basis for the science of which it treats. General practitioners, too, can study this book with great interest and profit.

---

A Manual of Hygiene. By Mary Taylor Bissell, Professor of Hygiene in the Woman's Medical College of the New York Infirmary for Women and Children. Octavo, cloth, $2.00. New York: The Baker & Taylor Co., 5 and 7 East Sixteenth street. 1894.

This excellent book should be in the hands of every householder. It contains just the sort of information that every intelligent person should possess. It is written by an experienced teacher, who is not pedantic, and, though prepared more particularly for medical students, it is, fortunately, adapted to a wider range of distribution and reading. The chapter on school hygiene deserves special mention, and merits the reading of every father and mother who have children of the school age.

We think this work merits an extensive sale, for the general public cannot have too much information on the subject of which it treats.

---

The Pocket Anatomist. By C. Henri Leonard, A. M., M. D., Professor of Gynecology, Detroit College of Medicine. Leather, 300 pages, 193 illustrations, post-paid, $1.00. Detroit, Mich.: The Illustrated Medical Journal Co., Publishers. 1894.

This little book has put in its annual appearance, this time, printed upon thin paper and bound in flexible red leather. This purports to be its eighteenth edition, and its make-up is such as to be particularly convenient for the pocket. It is founded upon

Gray's Anatomy, from which the engravings are copied. It is specially adapted to the use of students as a reminder, but in no way should supplant the use of complete anatomical works.

---

A SYNOPSIS OF THE PRACTICE OF MEDICINE. For Practitioners and Students. By WILLIAM BLAIR STEWART, A. M., M. D., Lecturer on Therapeutics; late Instructor on Practice of Medicine in the Medico-Chirurgical College of Philadelphia; Demonstrator in the Philadelphia School of Anatomy, etc. One large 8vo volume, about 434 pages; cloth, $2.75. New York: E. B. Treat, 5 Cooper Union. 1894.

This book has been given its appropriate title, for it is a *synopsis* of the practice of medicine; limited, however, to those diseases and conditions that do not come properly under the field of surgery, obstetrics or one of the specialties. The author announces his purpose as being to present to the profession a brief synopsis of the practice of medicine, but not to replace, by his work, those more extensive and elaborate publications which we term text-books. He has scanned all these, as well as current medical literature, with a view to obtain the best material from the most reliable sources. We are in sympathy with him when he asserts that the practice of medicine does not consist alone in the administration of drugs, but that it is a physician's duty to supervise the methods and habits of life of his patients in a physiological, hygienic and dietetic sense, as well as to control them by the influence of a firm mentality.

The publisher has made the book attractive and the author has made it useful within the limits of its sphere.

---

ESSENTIALS OF CHEMISTRY AND TOXICOLOGY. For the use of Students in Medicine. Twelfth edition. (Wood's Pocket Manuals.) By R. A. WITTHAUS, A. M., M. D., Professor of Medical Chemistry and Toxicology in the University of Vermont; Member of the Chemical Societies of Paris and Berlin, etc. 32mo, 314 pages, muslin, red edge; price, $1.00. New York: William Wood & Co. 1894.

The fact that eleven editions of this book have been exhausted testifies to its worth. In this edition, the part relating to the compounds of carbon has been, in great part, rewritten and rearranged to keep pace, as far as possible in a work of this character, with the advances in organic chemistry. The new chemical orthography adopted by the American Association for the Advancement of Science has been followed in this edition. To one unfamiliar with Prof. Witthaus's methods of teaching, it would be a surprise to witness the vast amount of valuable information upon the subject of medical chemistry that has been condensed into this small compass.

### BOOKS RECEIVED.

Diseases of the Ear. A Text-Book for Practitioners and Students of Medicine. By Edward Bradford Dench, Ph. B., M. D., Professor of Diseases of the Ear in the Bellevue Hospital Medical College ; Aural Surgeon, New York Eye and Ear Infirmary, etc., etc. Octavo, pp. xxii.—645. With eight colored plates and 152 illustrations in the text. New York : D. Appleton & Co. 1894.

Catalogue of Stoddart Bros., 84 Seneca street, Buffalo, N. Y., importers, manufacturers, wholesale and retail dealers in surgical instruments, trusses, braces, supporters, elastic stockings, batteries, crutches, operating tables, surgical chairs, orthopedic appliances, pharmaceutical preparations, strictly pure drugs and fine chemicals. Buffalo : Baker, Jones & Co. 1895.

Laboratory Guide for the Bacteriologist. By Langdon Frothingham, M. D. V., Assistant in Bacteriology and Veterinary Science, Sheffield Scientific School, Yale University. Illustrated. Price, 75c. Philadelphia : W. B. Saunders, 925 Walnut street. 1895.

Sexual Neurasthenia : Its Hygeine, Causes, Symptoms and Treatment, with a chapter on Diet for the Nervous. By George M. Beard, A. M., M. D., formerly Lecturer on Nervous Diseases in the University of the City of New York ; Fellow of the New York Academy of Medicine, etc., etc. Edited, with notes and additions, by A. D. Rockwell, A. M., M. D., formerly Professor of Electro-Therapeutics in the New York Post-Graduate Medical School and Hospital, etc., etc. With formulas. Fourth edition. Octavo, pp. xii.—294. Price, $2.75. New York : E. B. Treat, 5 Cooper Union. 1895.

Blood Serum Therapy and Antitoxins. By George E. Krieger, M. D., Surgeon to the Chicago Hospital, etc. Chicago : E. H. Colegrove & Co. 1895.

Syllabus of Gynecology, based on the American Text-Book of Gynecology. By J. W. Long, M. D., Richmond, Professor of Gynecology and Pediatrics in the Medical College of Virginia ; Gynecologist to the Hospital of the Medical College of Virginia ; Consulting Gynecologist to the Richmond City Dispensary, etc., etc. Price, $1.00 net. Philadelphia : W. B. Saunders, 925 Walnut street. 1895.

Notes on the Newer Remedies, their Therapeutic Applications and Modes of Administration. By David Cerna, M. D., Ph. D., Demonstrator of Physiology and Lecturer on the History of Medicine in the Medical Department of the University of Texas, formerly Assistant in Physiology, Demonstrator of, and Lecturer on, Experimental Therapeutics in the University of Pennsylvania, etc., etc. Small 8vo, pp. 253. Second edition, enlarged and revised. Price, $1.25. Philadelphia : W. B. Saunders, 925 Walnut street. 1895.

Surgical Pathology and Therapeutics. By John Collins Warren, M. D., Professor of Surgery, Medical Department Harvard University ; Surgeon to the Massachusetts General Hospital, etc. Octavo volume, pp. 832, with 135 illustrations. Price, cloth, $6.00 ; half-morocco, $7.00 net. Philadelphia : W. B. Saunders, 925 Walnut street. 1895.

